# Aerial drones for graft transport — ready for takeoff?

**DOI:** 10.3389/ti.2026.16512

**Published:** 2026-05-01

**Authors:** Benoit Mesnard, Joseph R. Scalea, Jaimeen Shah, Julien Branchereau

**Affiliations:** 1 Department of Urology and Transplantation Surgery, Nantes University Hospital, Nantes, France; 2 Nantes Université, CHU Nantes1, INSERM, Centre for Research in Transplantation and Translational Immunology, UMR 1064, ITUN5, Nantes, France; 3 Division of Transplantation, Department of Surgery, University of Maryland, Baltimore, MD, United States

**Keywords:** aircraft, organ transplantation, tissue and organ procurement, transportation, unmanned aerial vehicles

Dear editors,

The transport of grafts between the donor site and the recipient center remains a critical link in organ transplantation. It raises major organizational challenges and involves numerous professionals—physicians, nurses, procurement coordinators, and transport teams—and represents one of the determinants of cold ischemia time [1]. Cold ischemia itself is the most important modifiable predictor of short-, mid-, and long-term graft function. In kidney transplantation, cold ischemia is an independent factor significantly associated with delayed graft function [2] as well as long-term graft survival [3]. This issue is crucial, as it concerns not only the transport of organs from deceased donors but also potentially high-risk, high-stakes activities such as living-donor kidney transplantation, particularly within kidney exchange programs.

Organ transport currently relies on three main modalities: road networks using ambulances and taxis; rail transport; and air transport, often involving private jets. Aerial drones, and particularly uncrewed aircraft systems, defined as drones flown either by remote radio control or guided autonomously via GPS, could become an ideal solution in the future, as they offer several advantages.

Regarding clinical impact, long-distance ground transport may result in prolonged transport times. Ambulances are constrained by road networks and traffic conditions, while train transport depends on commercial lines and is therefore subject to scheduling constraints and limited flexibility. Organizational impact on clinical team: Automated drone transport offers improved logistical predictability and allows for around-the-clock organ reception. This increased fluidity could enable surgical teams to optimize operating room scheduling and reduce delays related to local logistics. Similarly, reducing the number of intermediaries involved in the transport of transplants is an important factor in minimizing logistical errors that may occur along the transportation chain. Public health and medicoeconomic impact: Drones represent a reliable, rapid, and less costly alternative to private jets and emergency road transport. Moreover, reductions in cold ischemia time, given its major prognostic value for short and long term graft function, carry significant medico-economic implications. Improved graft survival reduces the need for dialysis, which represents a major financial burden on healthcare systems. Environmental impact: Drone transport has a low carbon footprint compared with conventional air transport. Drones operate using either fully electric or hybrid propulsion systems. Their use could help reduce greenhouse gas emissions in alignment with ecological transition objectives. Occupational health: Replacing prolonged nighttime road trips (ambulance teams) with automated transport systems would reduce professional exposure to fatigue and accident risk while easing the logistical burden on on-call teams. In 2006, in France, two surgeons died in the crash of the aircraft that was transporting them to perform an organ procurement in a nearby city.

The first implementation of aerial drones for organ transport occurred in 2018 in Baltimore (USA) [4–6]. These early trials demonstrated stable thermal conditions during transport, reduced vibrations compared with traditional transport modes and minimized changes in spatial orientation. This work culminated in the first clinical kidney transplantation using drone transport, published in 2019, with an unremarkable postoperative course and good 30-day graft function. The drone used a six-rotor vertical propulsion system and flew at a cruising speed of approximately 32 km/h. These early experiments demonstrated technical feasibility but were constrained by the technological limitations of that time.

The use of uncrewed aircraft systems is now increasingly feasible thanks to technological advances. Current fully electric drone models achieve cruising speeds of around 100 km/h with ranges slightly surpassing 100 km. Larger hybrid-propulsion drones are more substantial models with wingspans exceeding 6 m, capable of reaching cruising speeds of approximately 120 km/h, ranges greater than 500 km, and payload capacities of around 40 kg. These drones, with increased payload capacity and volume, allow the use of clinically validated preservation containers, whether for static cold storage or hypothermic machine perfusion. Depending on the model, navigation may rely on radio-controlled guidance from a ground operator or on autonomous GPS-based flight along predefined trajectories. These devices represent the next-generation of drones, enabling transport with the lowest possible level of risk while minimizing the potential for human error. These new-generation fully autonomous drones are undergoing testing and have been evaluated in preclinical kidney transplantation experiments. In June 2025, the first preclinical experiments were conducted in France (Figure 1) using an allotransplantation model. Early results demonstrate stable thermal conditions and minimal vibration during 1-h transports, with further studies planned to confirm these findings over distances greater than 500 km.

**FIGURE 1:**
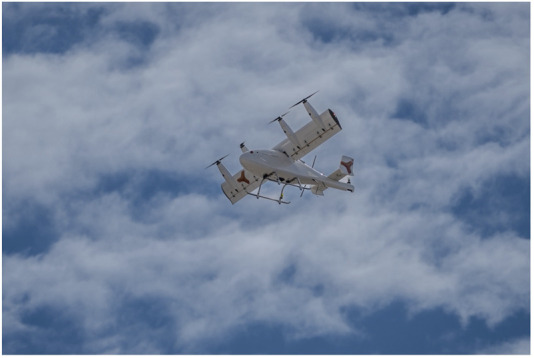
Fully electric aerial drone taking off for kidney graft transport during a preclinical experiment in France. The propulsion system consists of four rotors positioned vertically during takeoff and landing, which tilt horizontally during cruising flight. Image courtesy of Dufour Aerospace (Dübendorf, Switzerland).

These technological advances suggest that drone transport is becoming a mature modality for graft transportation, offering the technical possibility of connecting distant cities via direct fly routes [7]. While technological challenges are diminishing, new political and regulatory challenges are emerging. In most countries, drones intended for medical transport are regulated under civilian drone legislation. Numerous flight restrictions exist depending on population density, overflight of sensitive infrastructure, and proximity to critical zones. Establishing transfer routes between transplant centers requires the drafting and approval of flight plans meeting quality and safety standards equivalent to those of civil aviation. These regulations are nonetheless necessary and serve as a reminder that drones, by definition, are modes of transport more susceptible to hacking and diversion attempts.

Current regulations and the need to establish an aerial network dedicated to medical drone transport represent a major and labor-intensive challenge [8]. Clinical implementation will likely require future updates and adaptations to regulations specifically tailored to medical drone operations. Strong political engagement will be necessary to ease restrictions and enable the development of aerial drones for organ transport. It is up to us to make this technology the mode of organ transport for today and the future. The development of aerial drones could become a crucial logistical component in the future of organ preservation, particularly in the context of emerging normothermic perfusion hubs and the need for rapid transfer of grafts from the procurement site to strategic locations.

## Data Availability

The raw data supporting the conclusions of this article will be made available by the authors, without undue reservation.
